# Supplementary material for: Dynamic constriction and fission of endoplasmic reticulum membranes by reticulon
Source: Nat Commun. 2019 Nov 22;10:5327. doi: 10.1038/s41467-019-13327-7 (PMC6876568; doi:10.1038/s41467-019-13327-7)
Supplement: Supplementary file 1 — Supplementary Information [file 41467_2019_13327_MOESM1_ESM.pdf]

Dynamic constriction and fission of endoplasmic reticulum membranes by reticulon

Espadas et al.

Supplementary Information

Supplementary Figures

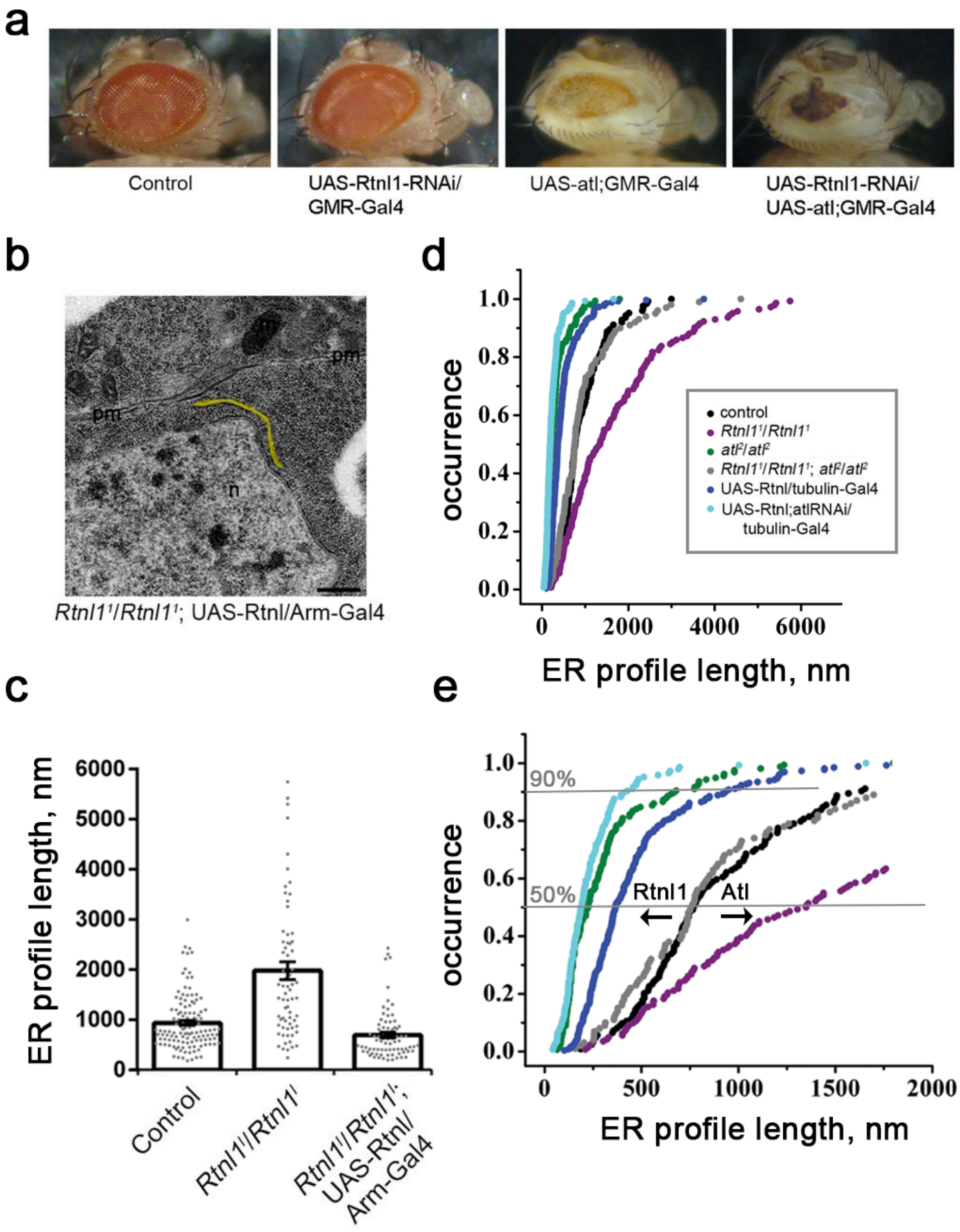

Supplementary Figure 1. a, Loss of *Rtnl1* via transgenic RNAi enhances the GMR- atlastin small eye phenotype. **b,** Rescue of *Rtnl1^1* mutants by re-expression of transgenic UAS-Rtnl1 under the control of the ubiquitous promoter armadillo-Gal4. ER profiles are highlighted. Scale bar 0.5 µm. pm, plasma membrane; n, nucleus. **c,** Average length of ER profiles measured on thin EM sections. Error bars represent S.E.M.; n>100. **d, e,** Cumulative distribution of the ER profile length for the indicated genotypes. e, Opposite effect of Atl and Rtnl1 on the median profile length (50%) and on long profiles (90%).

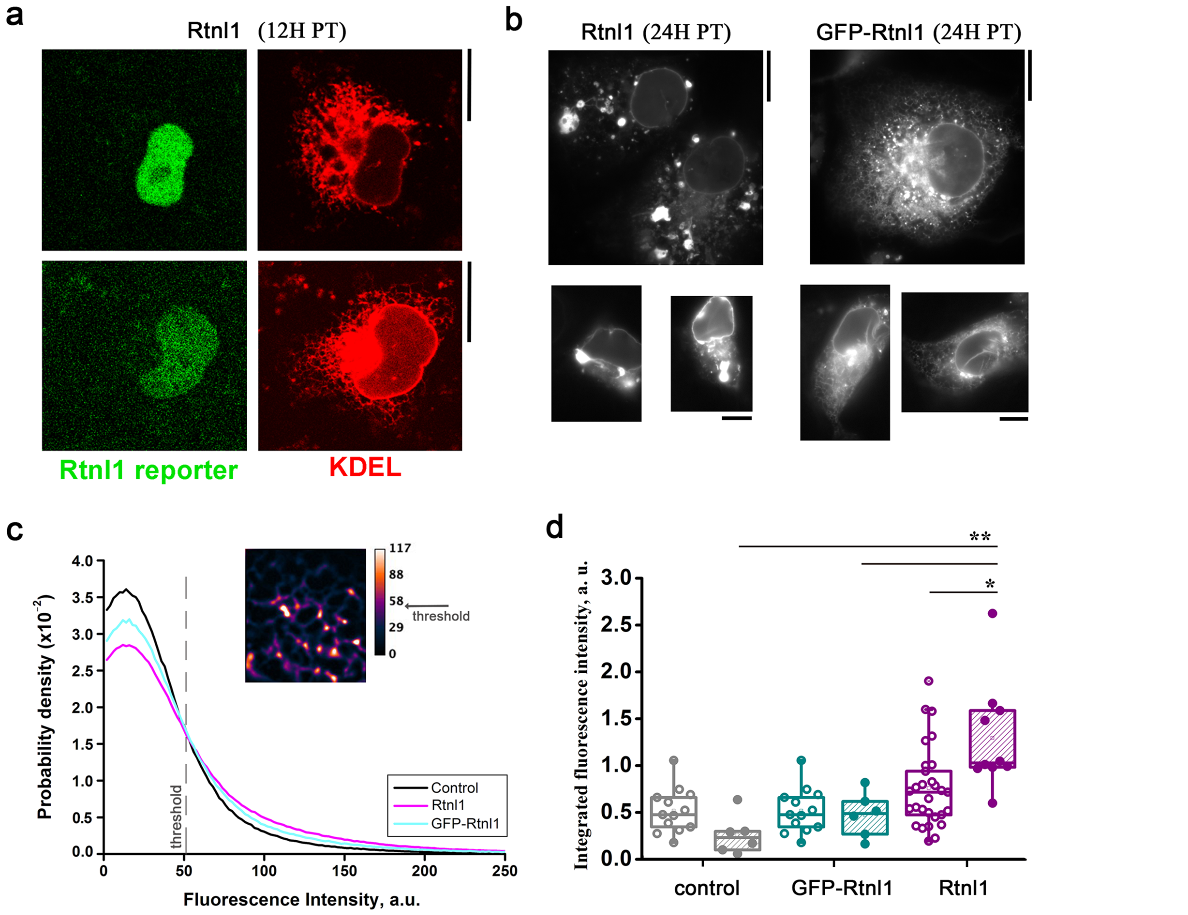

Supplementary Figure 2. Constriction and fragmentation of the ER network in COS-7 cells by Rtnl1 and GFP-Rtnl1. a, Constriction of the ER branches by Rtnl1 increases with Rtnl1 amount, identified by the simultaneous expression of nuclear CFP. The ER network is labeled by mCHERRY-KDEL, the two images illustrating high (upper panel) and low (lower panel) Rtnl1 expression were taken at 12H PT, scale bars 5 µm. **b,** Representative images of mCHERRY-KDEL-labeled ER network show that GFP-Rtnl1 is significantly impaired for transforming/fragmenting ER network in COS-7 cells. Images were taken at 24H PT, scale bars 5 µm. **c,** Probability densities of the mCHERRY-KDEL fluorescence intensity (per pixel, measured at 12H PT in COS-7 cells) measured for the conditions indicated (control-black, Rtnl1-magenta, GFP-Rtnl1-light blue) in randomly selected ROIs covering the peripheral ER (see Fig. 3a, Methods). Low intensity (below the threshold) correspond to the tubular ER network, while high intensities (above the threshold) correspond to bright fluorescent puncta emerging upon the ER constriction by Rtnl1 (insert, 24H PT, see also Fig. 3a of the main text). **d,** ER constriction measured as the increase of the integral fluorescence intensity above the threshold (shown in **c**) 12H (empty boxes) and 17H (dashed boxes) post-transfection in the control mCHERRY-KDEL (grey, n=13 cells @12H and n=5 cells @17H), GFP-Rtnl1+mCHERRY-KDEL (cyan, n=8 cells @12H and n=6 cells @17H) and Rtnl1+mCHERRY-KDEL (purple, n=12 cells @12H and n=8 cells @17H) cells. The protein expression level was estimated by GFP (GFP-Rtnl1) or CFP (Rtnl1-Mic, see **a**) fluorescence. Cells with the levels close to the mean value were selected for the analysis. One to three ROIs per cell were analyzed per each condition. Statistical significance: unpaired two-tailed *t*-test, $**p<0.01$, $*p<0.05$. Boxplots show IQR, with whiskers at 1.5IQR, square represents mean.

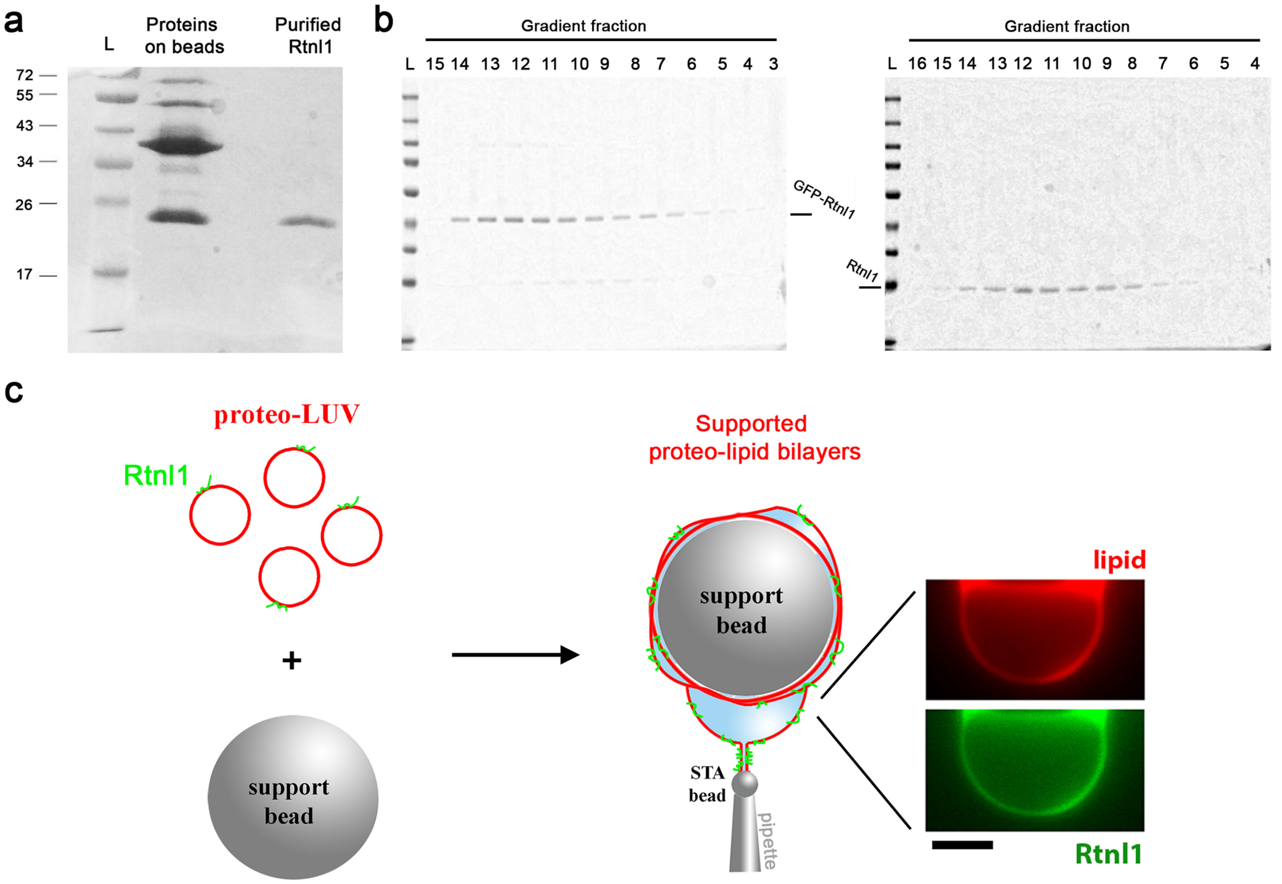

Supplementary Figure 3. Rtnl1 reconstitution into lipid nanotubes and nanotube-lamella sorting. a, SDS-PAGE gel showing different stages of Rtnl1 purification. **b**, SDS PAGE gels showing Rtnl1 and GFP-Rtnl1 incorporation into large unilamellar proteo-liposomes (proteo-LUVs, diameter 100nm). The proteo-LUVs were concentrated via liposome flotation assay (Methods), and the corresponding gradient fractions were collected and analysed. **c**, Proteo-LUVs were deposited on support beads to form proteo-lipid bilayers. The bilayers were doped with 0.2% of biotinylated lipids mediating membrane attachment to the 2 µm streptavidin-covered bead (STA-bead) suctioned into a micropipette tip. Membrane nanotubes were pulled from the reservoir membrane by increasing the distance between the support and the STA-bead. The fluorescence microphotographs show incorporation of Alexa488-Rtnl1 into the reservoir membrane. Scale bar 5 µm.

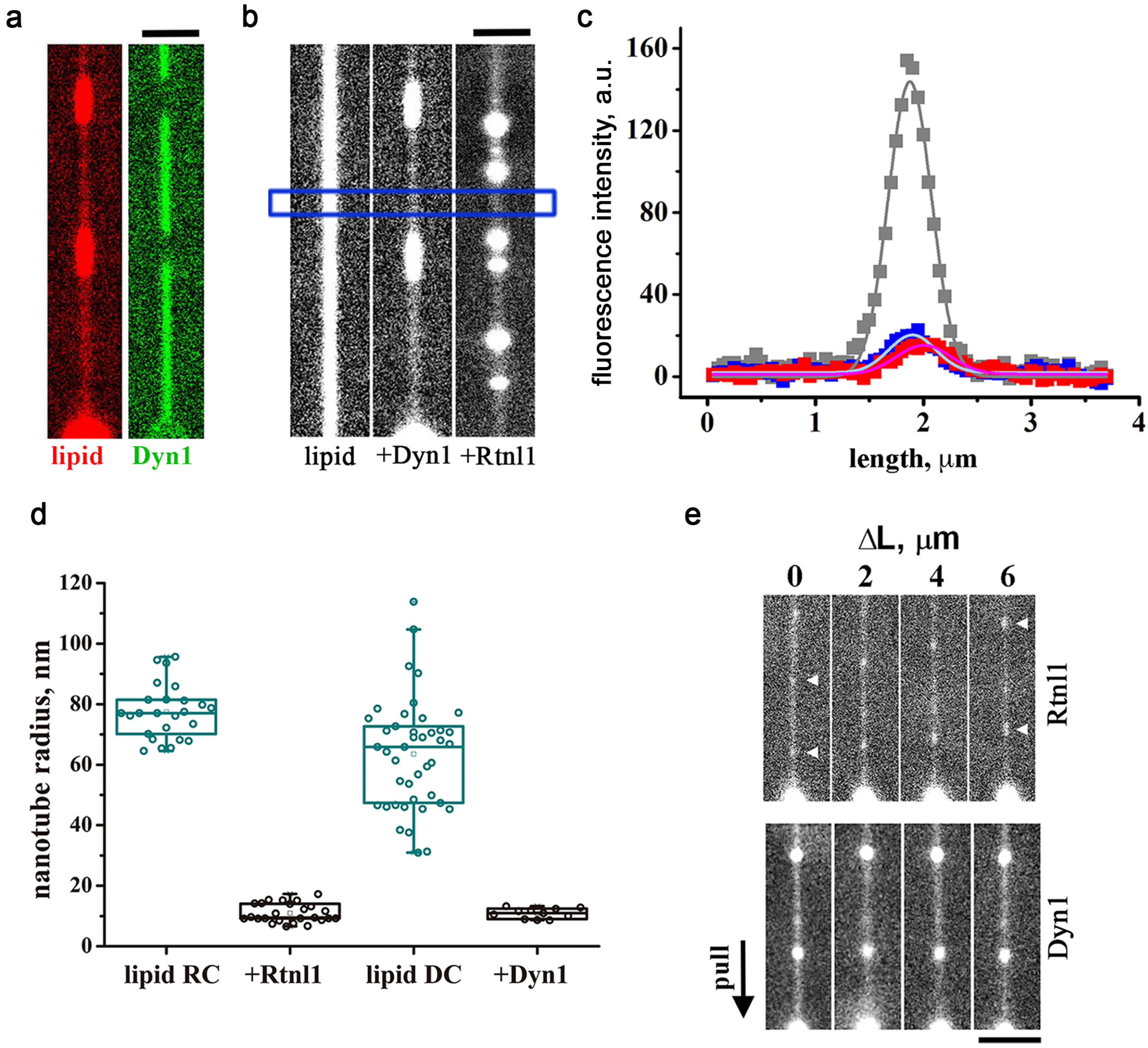

Supplementary Figure 4. Comparison of Rtnl1 and Dynamin1-driven nanotube constriction in static and dynamic tubes. a, Nanotube constriction by Dynamin1 labeled with Atto488 is shown. Dynamin1 (Dyn1, 0.5μM in the bulk) self-assembles into long cylindrical scaffolds (right). Their constriction activity is seen as the decrease of the fluorescence of the nanotube membrane labeled by Rh-DOPE (left), scale bar 5μm. **b,** Representative images of a pure lipid (lipid), Dynamin1-constricted (Dyn1) and Rtnl1-constricted (Rtnl1) nanotubes, Rh-DOPE fluorescence, is shown, scale bar 5μm. **c**, Fluorescence intensity profiles obtained from the nanotube cross-sections indicated by the blue rectangle in (**b**) (control-grey, Dyn1-blue, Rtnl1-red). **d,** Radii of pure lipid nanotubes (DC, n=43 tube, and RC, n=25 tubes, indicate Dyn1- and Rtnl1-specific lipid compositions respectively) and of the constricted regions of protein-containing nanotubes (n=11 tubes for Dyn1 and n=25 tubes for Rtnl1) obtained from fluorescence intensity (shown in **c**) as described in Methods. Boxplots show IQR, with whiskers at 1.5IQR, square represents mean. **e,** Representative image sequences showing Rtnl1- and Dyn1- constricted nanotubes during elongation. Parts of the tubes near the STA-bead (see Supplementary Fig. 2) are seen, scale bar 5μm.

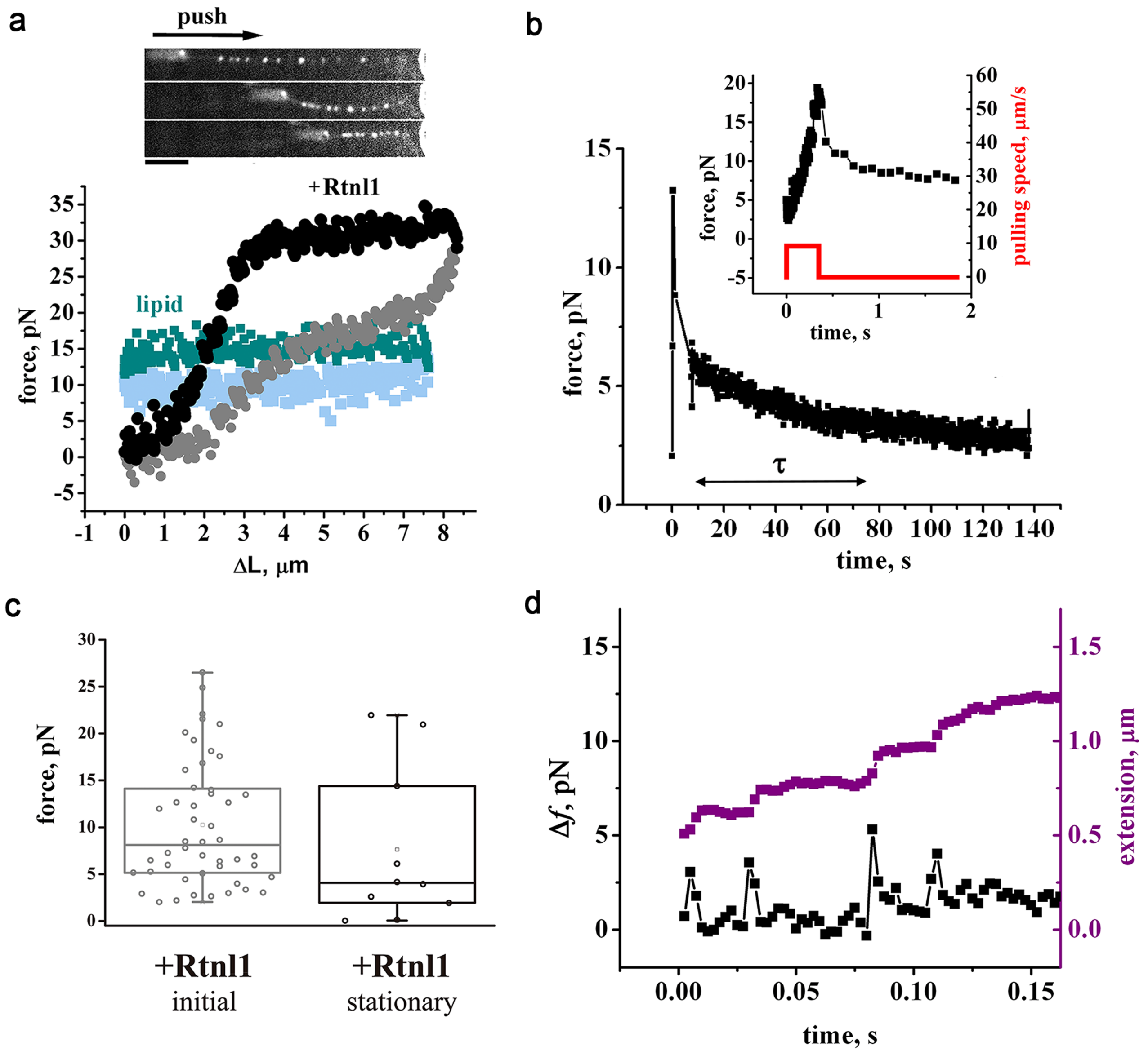

Supplementary Figure 5. Rtnl1 effects of the tensile force driving the nanotube pullback to the reservoir. a, Changes of the axial force during consecutive elongation/shortening (ΔL) of pure lipid (dark cyan/light blue) and Rtnl1- (black/grey) nanotubes at constant v_t=8μm/s. The image sequence shows sagging of Rtnl1-constricted membrane nanotube during shortening, Scale bar 5μm. **b,** Kinetics of the force reduction upon a brief step elongation of the tube (insert). The slow decrease (τ = 63.2 ± 8.7s) corresponds to the diffusional exchange of Rtnl1 between the nanotube and the reservoir ($D \sim L^2/\tau \sim 10^{-9}$cm^2/s). **c,** Axial force measured in Rtnl1-containing nanotubes before the diffusional relaxation (grey, n=48 tubes) and in the stationary state (black, n=8 tubes). Boxplots show IQR, with whiskers at 1.5IQR, square represents mean. **d,** Incremental pulling allows extending the Rtnl1-containing nanotube without lasting increase of the axial force. The stepwise slow extension (purple) causes only transient surges of the axial force (black), thus minimizing the dynamic stresses.

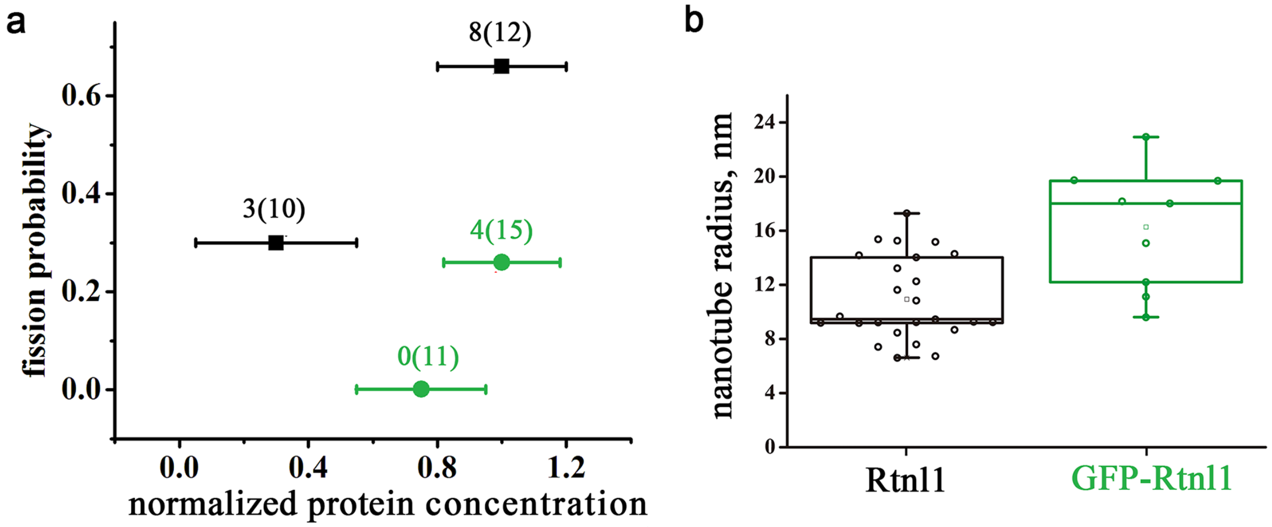

Supplementary Figure 6. Comparison of membrane fission activity of purified Rtnl1 and Rtnl1-GFP reconstituted into lipid nanotubes. a, Fission probability at different membrane concentrations of Rtnl1 and GFP-Rtnl1 (measured using flotation assay, as shown in Supplementary Fig. 3b, and normalized to the concentration obtained at 1:150 Rtnl1/lipid ratio). The nanotubes were pulled at v_t=20-30μm/s, the elongation length ΔL=35μm; #fissions(#tubes) are indicated above each data point on the graph. We note that fission probability surges when the protein concentration rises above some threshold protein level. Similar threshold-type behavior is observed in the ER fragmentation in COS-7 cells over-expressing Rtnl1 (see Supplementary Fig. 2d). **b,** Static (v_t=0) nanotube constriction by Rtnl1 (black, n=25 tubes) and GFP-Rtnl1 (green, n=9 tubes). Boxplots show IQR, with whiskers at 1.5IQR, square represents mean.

Supplementary Results

Theoretical analysis of membrane fission by Rtnl1

Curvature-driven sorting of Rtnl1

We consider a cylindrical proteo-lipid nanotube pulled from a low curved membrane bulging from membrane reservoir deposited on the bead surface[1] (Supplementary Fig. 3c). The free energy density (w) contains standard elastic ($\frac{1}{2}k\left(J-J_s\right)^2$) and mixing entropy ($\frac{k_BT}{a}\left[\varphi\ln\varphi+\left(1-\varphi\right)\ln(1-\varphi)\right]$ for the binary mixture of Rtnl1 and lipids) terms[2,3]. Here φ is the Rtnl1concentration in a membrane, measured as the area fraction $\varphi=\frac{a}{A+a}$, where $A+a$ is the membrane area per one Rtnl1 molecule and a is the area occupied by the molecule, $J_s = J_p\varphi$ is the intrinsic membrane curvature created by Rtnl1 at the concentration φ[4,5] (we assume that Rtnl1 molecules all have the same orientation dictated by their intrinsic curvature J_p, see below) and k is the mean curvature bending rigidity modulus, defined solely by the lipid component of the nanotube, as the nanotube coverage by Rtnl1 is sparse (see below). The Rtnl1 concentration in the nanotube differs from that in the reservoir due to curvature-composition coupling[6]. For small deviations δ we obtain:

$$(1)\ \ w=\frac{1}{2}k\left(J-J_p\varphi-\delta J_p\right)^2+\frac{k_BT}{2a}\frac{\delta^2}{\varphi\left(1-\varphi\right)}+\sigma$$

where σ is the lateral membrane tension and φ is the Rtnl1concentration in the membrane reservoir. From (1) we obtain the normalized deviation of the Rtnl1 concentration from its reservoir value (δ/φ) and the effective bending rigidity of the nanotube membrane (k_{eff})[7]:

$$(2)\ \ \frac{\partial w}{\partial\delta}=0\Rightarrow\frac{\delta}{\varphi}=\frac{\left(J-J_p\varphi\right)J_pa\left(1-\varphi\right)}{k_BT}k_{eff}$$

$$(3)\ \ \frac{\partial^2 w}{\partial J^2}=k_{eff}=\frac{k}{1+\frac{kJ_p^2a\varphi\left(1-\varphi\right)}{k_BT}}$$

We note that k_{eff} is smaller than k, explaining facilitation of nanotube constriction by Rtnl1 (Fig. 4c). Substituting (2) and (3) into (1) we obtain the free energy of a stationary cylindrical membrane nanotube:

$$(4)\ \ W=\left(\frac{1}{2}k_{eff}\left(J-J_p\varphi\right)^2+\sigma\right)\frac{2\pi L}{J}-fL$$

where f is the axial force (f_p in Fig. 4a). Minimizing (4) with respect to J and L we obtain the stationary force and radius of the nanotube (R_t)[7]:

$$(5)\ \ R_t=\sqrt{\frac{k_{eff}}{2\left(\sigma+\frac{1}{2}k_{eff}J_p^2\varphi^2\right)}}=\sqrt{\frac{k_{eff}}{2\sigma_0}}$$

(6) $f = 2\pi\left(\sqrt{2k_{eff}\sigma_0} - k_{eff}J_p\varphi\right)$

Combining (5) and (6) yields:

(7) $f = 2\pi k_{eff}\left(\frac{1}{R_t} - \varphi J_p\right)$

which for a pure lipid nanotube recovers[3]:

(7a) $f = \frac{2\pi k_l}{R_t}$

Equations (7a) was used to calculate k_l using stationary values of f and R_t measured experimentally. At v_t=0, $k_l = 0.75\cdot10^{-19}J$ was obtained, consistent with published data[8]. From (2) and (3) we further obtain the sorting coefficient χ characterizing Rtnl1 distribution between the low (here we assume J=0) and highly curved membranes (Fig. 4d):

(8) $\chi = \frac{\varphi\left(J = \frac{1}{R_t}\right)}{\varphi\left(J=0\right)} - 1 = \frac{J_p a\left(1-\varphi\right)k_l}{R_t k_B T}$

and the corresponding change in the bending rigidity (Fig. 4c):

(9) $\frac{\Delta k}{k_{eff}} = \chi R_t \varphi J_p = \frac{aJ_p^2\varphi\left(1-\varphi\right)k_l}{k_B T}$

For GFP-Rtnl1 $\chi = 1.5$ was calculated directly (using the data shown in Fig. 4c). Assuming $\varphi \ll 1$ we found aJ_p = 1.33 nm. To estimate χ for non-labeled Rtnl1 we analyze the force relaxation upon a brief elongation step (Supplementary Fig. 5b,c). Considering that the protein lateral diffusion is slow, the *initial* force (f_0) is:

(10) $f_0 = 2\pi k_l\left(\frac{1}{R_t}\sqrt{\frac{k_{eff}}{k_l}} - \varphi J_p\right)$

while the final (*stationary*) force is defined by Eq.7. We rewrite (7) and (10) to make a system of two equations

(10a) $\begin{cases} \varphi J_p = \frac{1}{R_{Rtnl1}} - \frac{f_{Rtnl1}}{2\pi k_{eff}} \\ \frac{f_{Rtnl1}}{2\pi k_{eff}} - \frac{f_0}{2\pi k_l} = \frac{1}{R_{Rtnl1}}\left(1 - \sqrt{\frac{k_{eff}}{k_l}}\right) \end{cases}$

with two unknown variables k_{eff} and φJ_p. Using the experimentally measured values of the *initial* and *stationary* forces (from Supplementary Fig. 5c), the nanotube radius (from Fig. 4b, 1:150 Rtnl1/lipid), and the bending rigidity of the lipid bilayer (from Fig. 4c), we obtained $k_{eff} = 0.2\cdot10^{-19}J$ and $\varphi J_p = 0.023nm^{-1}$. The latter is equal to the spontaneous curvature of the reservoir membrane J_s. It is notably close to the curvature of LUVs used for Rtnl1 reconstitution (0.02nm^{-1}, Supplementary Fig. 3c). This

closeness strongly indicates that Rtnl1 would prefer a single membrane orientation in the proteo-LUVs, and hence in the reservoir membranes, with its cytoplasmic parts facing the vesicle exterior.

Using Eq. 9 we found that the calculated sorting coefficient for unlabeled Rtnl1 is substantially (5-6 folds) higher than $\chi_{GFP-Rtnl1}$ directly measured for GFP-Rtnl1 (see Methods). Crucially, the discrepancy is eliminated if we consider Rtnl1 oligomerization index be larger than GFP-Rtnl1 one. Assuming that GFP binding to Rtnl1 has no effect on its membrane wedging activity (on J_p) and only affects the protein oligomerization, and considering that for 1:150 Rtnl1/lipid ratio $\frac{a(1-\varphi)}{\varphi} = A \approx 50nm^2$, we find by combining (8) and (10a) the protein membrane coverage in the reservoir to be φ =0.13 (consistent with close-to-complete incorporation of the proteins into proteo-liposomes seen by flotation assays, see Methods) and the protein parameters J_p=0.17nm^{-1} and a=7.8 nm^2, consistent with the published values[5]. This consistency corroborates the notion that GFP impairs oligomerization of GFP-Rtnl1.

Dynamic membrane constriction by Rtnl1

Outline of the model. Here we consider the same cylindrical proteo-lipid nanotube as above, pulled from the (quasi)planar membrane at a constant velocity v_t (Fig. 5a). We assume that Rtnl1 augments the surface viscosity of the planar membrane to its concentration. We further assume that during the nanotube elongation Rtnl1 migrates together with lipids to minimize friction losses. The migration is coupled to curvature-driven sorting of Rtnl1. As the Rtnl1 concentration in the nanotube is higher than in the reservoir membrane (Fig. 4d), the nanotube elongation creates a diffusion flux in the reservoir membrane towards the nanotube, causing Rtnl1 depletion in the reservoir membrane near the nanotube. The Rtnl1 depletion diminishes the integral viscous resistance to pulling, explaining the shear-thinning effect observed experimentally (Fig. 5d).

Protein transport equation. Rtnl1 flux I in the tube is:

(11) $$I = 2\pi R_t v_t c_t$$

where c_t is the Rtnl1 concentration in the nanotube and R_t is the nanotube radius (we assume that the nanotube preserves cylindrical shape during elongation). The protein flux in the reservoir membrane is defined by the gradient of Rtnl1 concentration $c(r)$:

(12) $$2\pi r D \frac{dc}{dr} + 2\pi r v c = I$$

From continuity of the membrane

(13) $$v(r) r = v_t R_t$$

we obtain:

(14) $$\frac{dc}{dr} + \frac{v_t R_t}{D} \frac{(c - c_t)}{r} = 0$$

Assuming $\frac{v_t R_t}{D} \ll 1$, the solution of (14) is:

$$(15)\, c(r) = c_0 + (c_t - c_0)\left[1 - \left(\frac{R}{r}\right)^{\frac{v_t R_t}{D}}\right] \sim c_0 + (c_t - c_0)\frac{v_t R_t}{D}\ln\frac{R}{r}$$

where R is the reservoir size (e.g. the diameter of the support bead) and c_0 – is the protein concentration in the planar membrane

Sorting in elongating tubes. Eq. (8) describes the linear sorting regime for static nanotubes. As elongating nanotubes are significantly narrower, we resort to non-linear approximation[9]:

$$(16)\ c_t = c(R_t)\exp\left[\frac{\Delta w}{k_B T}\right]$$

where $\Delta w \approx \frac{k_l J_p a}{2R_t}$ is the energy gain (per molecule) in Rtnl1 transfer from a planar to curved nanotube membrane (note that linear expansion of (16) recovers (8) for small φ). From 15 and 16 we obtain:

$$(17)\ c_t = c_0 \exp\left(\frac{R_j}{R_t}\right)\frac{\frac{v_t}{v_D}+1}{\exp\left(\frac{R_j}{R_t}\right)\frac{v_t}{v_D}+1}$$

and

$$(17a)\ c(R_t) = c_0\frac{\frac{v_t}{v_D}+1}{\exp\left(\frac{R_j}{R_t}\right)\frac{v_t}{v_D}+1}$$

where $D_1 = \frac{D}{\ln\frac{R}{R_t}}$ is a renormalized diffusion coefficient (further assumed constant), $v_D = \frac{D_1}{R_t}$ and $R_J = \frac{k_l J_p a}{2k_B T}$ is a characteristic length associated with sorting. Note that from (17) it follows that at large v_t the sorting is inhibited as c_t approaches c_0.

Axial force and constriction. Eq. (7) for stationary force (v_t=0) can be modified to describe the "elastic" extension (corresponding to the linear force growth, Fig 5a, c):

$$(18)\, \Delta f = 2\pi k_l \Delta\left(\frac{1}{R_t}\right)$$

Eq. (18) was used to recalculate membrane constriction (Fig 5e) from the force values obtained experimentally at different pulling rates (Fig 5d).

Viscous friction and pulling force. The work produced by the pulling force $f \cdot v_t$ goes to frictional dissipation Q and changes of W.

(19) $$\frac{dW}{dt} + Q = fv_t$$

Dissipation in the planar part is calculated as[10,11]:

(20) $$Q = \int_{R_t}^{R} \eta(r) \left(\frac{v_t R_t}{r^2} \right)^2 2\pi r dr$$

Dissipation in the nanotube can be neglected for sufficiently long tubes. In the leading order, the surface viscosity depends linearly on Rtnl1 concentration:

(21) $$\eta(r) = \eta_l (1 - ac) + \eta_p ac \approx \eta_l + \eta_p ac$$

Hence:

(22) $$Q = m_l v_t^2 + m_p(v_t) v_t^2$$

where m_l is the "lipid" friction $m_l = 4\pi\eta_l$ and m_p is the protein one:

(23) $$m_p = 4\pi\eta_p ac_0 \frac{\frac{v_t}{v_D} + 1}{\exp\left(\frac{R_j}{R_t}\right)\frac{v_t}{v_D} + 1}$$

Collecting the above, we obtain:

(24) $$f_0^2 = f\left(f - 2\left(m_l + m_p(v_t)\right)v_t\right)$$

where $f_0^2 = 8\pi^2 k_l \sigma$ characterizes the reservoir lateral tension.

In pure lipid nanotube at low speeds $f_0 \sim f$ so that

(25) $$(f - f_0) = 4\pi\eta_l v_t$$

From (25) the viscosity η_l is estimated as $2 \cdot 10^{-6}$g/cm[10,11].

In Rtnl1-containing tubes f at the point of fission was always much higher than f_0. In this case, (24) can be simplified so that Rtnl1-specific contribution to viscous resistance becomes:

(26) $$f = 2m_p(v_t, R_t)v_t$$

We further note that in our experiments $\exp\left(\frac{R_J}{R_t}\right)\frac{v_t}{v_D} \gg 1$ so that, upon subtraction of the pure lipid contribution, (23) and (26) can be combined to obtain:

(27) $$f_{Rtnl1} \exp\left(\frac{f_{Rtnl1}}{f_s}\right) = 4\pi\eta_p ac_0 (v_t + v_D)$$

where $f_s = \frac{4\pi k_B T}{J_p a}$ is the effective "sorting" force. At large v_t, it follows from Eq. 27 that $f_{Rtnl1} \sim \ln\left(v_t\right)$.

Equation (27) was used to fit the Rtnl1-specific contribution to friction resistance (Fig. 4d, insert) and obtain the effective viscosity due to Rtnl1 $\eta_p a c_0$.

Creation versus scission of membrane tubules by Rtnl1

The above theoretical analyses reveal that in both static (v_t=0) and dynamic (v_t>0) nanotubes the increase of Rtnl1 concentration in the nanotube membrane leads to more curvature. The situation with the axial force is different: Rtnl1 decreases the static force (Supplementary Fig. 5a-c, in agreement with stabilization of tubes and tubular networks) and increases the force required to pull the tube (Fig. 5c,d). Such an increase of the force barrier and the associated stochastic destabilization of the nanotubes are seemingly in contrast with the ER curvature creation/stabilization by reticulons. However, we note that the force develops gradually with the nanotube extension. At the beginning of the pulling, the force is smaller than that seen in a pure lipid system (Supplementary Fig. 5a), hence initially Rtnl1 always facilitates the tube creation. It takes time/extension to reach forces associated with substantial curvature stress and fission (Fig. 5c, Supplementary Fig. 5a). At low, physiologically relevant v_t, the stress is not high, and fission is probabilistic. Upon finishing the elongation, the stress dissipates (Supplementary Fig. 5b), bringing the fission probability effectively to zero. Finally, incremental, small-step elongation of the nanotube can be performed with only minimal and transient stress increase (Supplementary Fig. 5d). We conclude that the dynamic friction-driven membrane constriction by Rtnl1 can be controlled separately from its curvature creation/tubulation activities.

Kinetic model of Atl-Rtnl1 interactions in ER maintenance

The controlled separation of creative and destructive activities of Rtnl1 becomes even more important in the context of its interactions with Atl in the ER maintenance and remodeling. Without going into mechanistic details of these interactions (subject of further research), we propose a rudimentary kinetic model for *qualitative* summarizing of our experimental observation of Atl-Rtnl1 functional antagonism. The model is based on the following assumptions:

- Rtnl1 and Atl work *synergistically* to incorporate new branches (connections) into the ER network. Here Rtnl1 facilitates curvature production and also the extension of the branches by molecular motors and microtubules via reduction of the initial force barrier. Atl, in turn, mediates the incorporation of newly formed branches into the network: without Atl-mediated fusion the branches retract. In the most general terms the incorporation goes proportionally to $K_1 c_{Atl}^{\alpha}\left(M + K_2 c_{Rtnl1}^{\beta}\right)$, where c_{Atl} and c_{Rtnl1} correspond to the membrane concentrations of Atl and Rtnl1 (to the power of α and β), α and β indicate the number of Atl and Rtnl1 molecules involved in an incorporation of a single branch, K_1 and K_2 are kinetic constants, and M accounts for the other factors involved in the branch formation, e.g. pulling by molecular motors.

- Rtnl1 acts *antagonistically* to the above incorporation process by occasionally breaking the incorporated branches via velocity-dependent membrane fission; in the most general terms, the breakage probability is proportional to $K_3\left(v_t\right)c_{Rtnl1}^{\beta+\gamma}$, where γ is positive reflecting higher cooperativity index of fission as compared to tubulation.

The two processes define the turnover of the branches as:

$$(28)\ \frac{dn}{dt}=\left(K_1 c_{Atl}^{\alpha}\left(M+K_2 c_{Rtnl1}^{\beta}\right)n^2\right)\left(1-\frac{n}{n_{\max}}\right)-K_3\left(v_t\right)c_{Rtnl1}^{\beta+\gamma}n$$

where n is the total number of the branches in the network and n_{max} is the maximum number of connections defined by material/physical limitations. In the stationary situation (for $n \ll n_{max}$):

$$(29)\ n=\frac{K_3\left(v_t\right)c_{Rtnl1}^{\beta+\gamma}}{K_1 c_{Atl}^{\alpha}\left(M+K_2 c_{Rtnl1}^{\beta}\right)}\approx\Omega\frac{c_{Rtnl1}^{\gamma}}{c_{Atl}^{\alpha}}$$

Eq.(29) summarizes the functional antagonism between the two proteins. The ER profile length $L\sim\frac{L_{\max}}{n}\sim\frac{c_{Atl}^{\gamma}}{c_{Rtnl1}^{\alpha}}$ increases with Atl and decreases with Rtnl1 as observed experimentally (Fig. 2c, Supplementary Fig. 1d, e).

References

1 Velasco-Olmo, A., Ormaetxea Gisasola, J., Martinez Galvez, J. M., Vera Lillo, J. & Shnyrova, A. V. Combining patch-clamping and fluorescence microscopy for quantitative reconstitution of cellular membrane processes with Giant Suspended Bilayers. *Scientific reports* 9, 7255, doi:10.1038/s41598-019-43561-4 (2019).

2 Sorre, B. *et al.* Curvature-driven lipid sorting needs proximity to a demixing point and is aided by proteins. *Proceedings of the National Academy of Sciences of the United States of America* 106, 5622-5626, doi:10.1073/pnas.0811243106 (2009).

3 Tian, A. & Baumgart, T. Sorting of lipids and proteins in membrane curvature gradients. *Biophysical journal* 96, 2676-2688, doi:10.1016/j.bpj.2008.11.067 (2009).

4 Brady, J. P., Claridge, J. K., Smith, P. G. & Schnell, J. R. A conserved amphipathic helix is required for membrane tubule formation by Yop1p. *Proceedings of the National Academy of Sciences of the United States of America* 112, E639-648, doi:10.1073/pnas.1415882112 (2015).

5 Hu, J. *et al.* Membrane proteins of the endoplasmic reticulum induce high-curvature tubules. *Science* 319, 1247-1250, doi:10.1126/science.1153634 (2008).

6 Callan-Jones, A., Sorre, B. & Bassereau, P. Curvature-driven lipid sorting in biomembranes. *Cold Spring Harbor perspectives in biology* 3, doi:10.1101/cshperspect.a004648 (2011).

7 Capraro, B. R., Yoon, Y., Cho, W. & Baumgart, T. Curvature sensing by the epsin N-terminal homology domain measured on cylindrical lipid membrane tethers. *Journal of the American Chemical Society* 132, 1200-1201, doi:10.1021/ja907936c (2010).

8 Shi, Z. & Baumgart, T. Membrane tension and peripheral protein density mediate membrane shape transitions. *Nature communications* 6, 5974, doi:10.1038/ncomms6974 (2015).

9 Aimon, S. *et al.* Membrane shape modulates transmembrane protein distribution. *Developmental cell* 28, 212-218, doi:10.1016/j.devcel.2013.12.012 (2014).

10 Brochard-Wyart, F., Borghi, N., Cuvelier, D. & Nassoy, P. Hydrodynamic narrowing of tubes extruded from cells. *Proceedings of the National Academy of Sciences of the United States of America* 103, 7660-7663, doi:10.1073/pnas.0602012103 (2006).

11 Chizmadzhev, Y. A. *et al.* Lipid flow through fusion pores connecting membranes of different tensions. *Biophysical journal* 76, 2951-2965, doi:10.1016/S0006-3495(99)77450-3 (1999).
