# Supplementary material for: Dynamic constriction and fission of endoplasmic reticulum membranes by reticulon
Source: Nat Commun. 2019 Nov 22;10:5327. doi: 10.1038/s41467-019-13327-7 (PMC6876568; doi:10.1038/s41467-019-13327-7)
Supplement: Supplementary file 3 — Description of Additional Supplementary Files [file 41467_2019_13327_MOESM3_ESM.docx]

**Description of Additional Supplementary Files**

File Name: Supplementary Movie 1

Description: Stack of optical sections through an atl2 neuron. Scale bar 200nm.

File Name: Supplementary Movie 2

Description: 3D reconstruction of atl2 neuronal ER from movie 1 stack. Scale bar 200nm.

File Name: Supplementary Movie 3

Description: Stack of optical sections through an Rtnl11 /atl2 neuron. Scale bar 200nm.

File Name: Supplementary Movie 4

Description: 3D reconstruction of Rtnl11 /atl2 neuronal ER from movie 3 stack. Scale bar 200nm.

File Name: Supplementary Movie 5

Description: Stack of optical sections through a control neuron. Scale bar 200nm.

File Name: Supplementary Movie 6

Description: 3D reconstruction of control neuronal ER from movie 5 stack. Scale bar 200nm.

File Name: Supplementary Movie 7

Description: Stack of optical sections through an Rtnl11 neuron. Scale bar 200nm.

File Name: Supplementary Movie 8

Description: 3D reconstruction of Rtnl11 neuronal ER from movie 7 stack. Scale bar 200nm

File Name: Supplementary Movie 9

Description: Example of scission of individual ER branches in COS-7 cells overexpressing Rtnl1. The

movie was taken at 10 frames/s. The frame sequence corresponding to Movie 9 is shown in Fig. 3c.

File Name: Supplementary Movie 10

Description: Example of scission of individual ER branches in COS-7 cells overexpressing Rtnl1. The

movie was taken at 10 frames/s.

File Name: Supplementary Movie 11

Description: Example of scission of individual ER branches in COS-7 cells overexpressing Rtnl1. The

movie was taken at 10 frames/s.

File Name: Supplementary Movie 12

Description: Fission of a nanotube pulled from Rtnl1-containing membrane reservoir. The movie

was recorded at 30 frames/s.

File Name: Supplementary Movie 13

Description: Fission of a nanotube pulled from Rtnl1-containing membrane reservoir. The movie

was recorded at 10 frames/s. The frame sequence corresponding to the Movie13 is shown in Fig. 5b.
